# Therapeutic Effects of microRNAs on Nonalcoholic Fatty Liver Disease (NAFLD) and Nonalcoholic Steatohepatitis (NASH): A Systematic Review and Meta-Analysis

**DOI:** 10.3390/ijms24119168

**Published:** 2023-05-23

**Authors:** Yuezhi Zhu, Jen Kit Tan, Sok Kuan Wong, Jo Aan Goon

**Affiliations:** 1 Department of Biochemistry, Faculty of Medicine, Universiti Kebangsaan Malaysia, Kuala Lumpur 56000, Malaysia; 2 Department of Pharmacology, Faculty of Medicine, Universiti Kebangsaan Malaysia, Kuala Lumpur 56000, Malaysia

**Keywords:** NAFLD, NASH, miRNAs, therapeutic effects

## Abstract

Nonalcoholic fatty liver disease (NAFLD) has emerged as a global health problem that affects people even at young ages due to unhealthy lifestyles. Without intervention, NAFLD will develop into nonalcoholic steatohepatitis (NASH) and eventually liver cirrhosis and hepatocellular carcinoma. Although lifestyle interventions are therapeutic, effective implementation remains challenging. In the efforts to establish effective treatment for NAFLD/NASH, microRNA (miRNA)-based therapies began to evolve in the last decade. Therefore, this systematic review aims to summarize current knowledge on the promising miRNA-based approaches in NAFLD/NASH therapies. A current systematic evaluation and a meta-analysis were conducted according to the PRISMA statement. In addition, a comprehensive exploration of PubMed, Cochrane, and Scopus databases was conducted to perform article searches. A total of 56 different miRNAs were reported as potential therapeutic agents in these studies. miRNA-34a antagonist/inhibitor was found to be the most studied variant (*n* = 7), and it significantly improved the hepatic total cholesterol, total triglyceride, Aspartate Aminotransferase (AST), and Alanine Transaminase (ALT) levels based on a meta-analysis. The biological processes mediated by these miRNAs involved hepatic fat accumulation, inflammation, and fibrosis. miRNAs have shown enormous therapeutic potential in the management of NAFLD/NASH, wherein miRNA-34a antagonist has been found to be an exceptional potential agent for the treatment of NAFLD/NASH.

## 1. Introduction

Nonalcoholic fatty liver disease (NAFLD) is a condition in which excess fat is built up in the liver. NAFLD refers to a spectrum of conditions that ranges from simple fatty liver to nonalcoholic steatohepatitis (NASH). When too much fat is accumulated in the liver as a result of overweight, adiposity, insulin resistance, and metabolic dysfunction, at least 5% of the liver tissues will develop steatosis. Inflammation of the hepatic lobules and progressive NASH with or without fibrosis will eventually lead to liver failure, hepatic cirrhosis, and hepatocellular carcinoma [1,2,3]. NAFLD influences 30–40% of the US population, 20–44% of the European population, 29.6% of the Asian population, and approximately 45% of Hispanics. The prevalence of NAFLD in children has also increased annually in the last few years, with a growth rate of about 8–11%, and children with obesity having a prevalence of over 38% [2]. Urbanization, a westernized diet, and a sedentary and physically inactive lifestyle are the leading causes of NAFLD [2,4,5].

Recent studies have concluded that NAFLD is causally related to both metabolic syndrome and type 2 diabetes. Together these contribute to the development of cardiovascular disease, chronic kidney disease, and a multitude of extrahepatic neoplasms [1,2,6,7]. As research continues in other directions, including the pathogenesis and clinical features of NAFLD, NAFLD is thought to be highly heterogeneous, with significant variation in the rate of progression of the disorders and response to therapy [8]. As such, an international group of experts suggested to modify the name of NAFLD to “Metabolic Dysfunction-Associated Fatty Liver Disease” (MAFLD) in the year 2020 [8]. However, irregularities between the criteria for NAFLD and MAFLD diagnosis prohibited the change [9,10]. Much effort has been taken to develop treatments for NAFLD/NASH because lifestyle interventions, although curative, are challenging to implement effectively and adhere to over the long term [4,11,12,13,14]. However, NAFLD/NASH is at risk of developing into cirrhosis and hepatocellular carcinoma, and no drugs are currently recognized as a cure.

The dysregulation of microRNA (miRNA) has been implicated as a causative factor for the development of NAFLD. miRNA is a family of small endogenous RNAs, made up of 15–25 nucleotides, known to modulate cell proliferation, differentiation, and apoptosis at the post-transcriptional level. They have been closely linked to several physiological and pathophysiological processes, including metabolism, immunity, and neoplasms in the human body [15,16]. Studies have demonstrated that miRNAs are differentially expressed in normal subjects and patients with NAFLD/NASH. Some of these miRNAs may be implicated in the pathophysiological processes of NAFLD/NASH by dysregulating lipid metabolism, cell proliferation, inflammation, apoptosis, cell cycle, and fibrosis [16]. In addition, some studies have shown that miRNA expression can reduce triglyceride accumulation and inhibit adipocyte differentiation, indirectly regulating the body’s lipid metabolism, including triglyceride and HDL binding, cholesterol transport, and fatty acid synthesis and oxidation involving lipid metabolizing enzymes [17,18,19]. miRNAs can promote or inhibit the development of hepatocellular carcinoma (HCC) in patients with NAFLD/NASH, and miRNAs in HCC-derived exosomes can be used to diagnose HCC [20]. Because miRNAs control post-transcriptional modification and gene expression and are incredibly stable, miRNAs are increasingly employed in disease detection and prediction, disease progress monitoring, and disease therapy [21].

Over the last few decades, much research has demonstrated that activation or inhibition of miRNAs can control the development of NAFLD/NASH. With the increasing number of cellular or animal models, preclinical validation of miRNA for therapeutic purposes has become increasingly refined. Here, we provide a systematic review and meta-analysis to summarize the results of miRNA-based therapeutic approaches in cellular or animal models of NAFLD/NASH, and comprehensively discuss the overall efficacy of miRNAs as a therapeutic agent of NAFLD/NASH.

## 2. Results and Discussion

### 2.1. Results

#### 2.1.1. Study Characteristics

**Figure 1:**
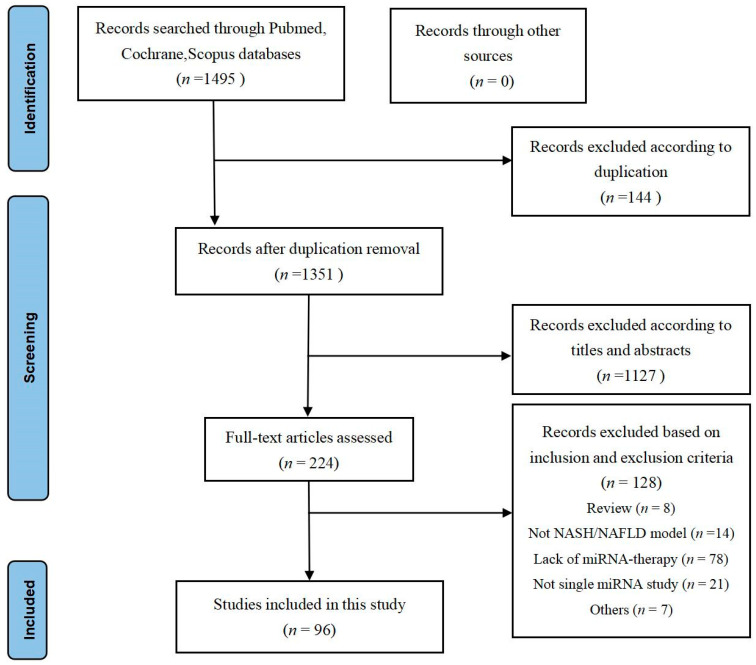
PRISMA flowchart. The final 96 research studies that met the criteria were included in the qualitative synthesis based on the number of titles, abstracts, and whole texts screened.

A search of the databases determined 1495 titles with abstracts (Figure 1). After preliminary screening, 1271 articles were excluded based on duplication (144) and title abstract screening (1127), and the remainder 224 articles were reviewed for both full texts. Consequently, 128 were excluded because they were: review articles (*n* = 8), not including an NASH/NAFLD model (*n* = 14), lacking miRNA therapy (*n* = 78), not using a single miRNA (*n* = 21), or other reasons including lack of assessment of therapeutic effect and no relevant outcomes reported (*n* = 7). Finally, 96 articles met the inclusion criteria and were incorporated into this review. The detailed characteristics of these 96 [19,22,23,24,25,26,27,28,29,30,31,32,33,34,35,36,37,38,39,40,41,42,43,44,45,46,47,48,49,50,51,52,53,54,55,56,57,58,59,60,61,62,63,64,65,66,67,68,69,70,71,72,73,74,75,76,77,78,79,80,81,82,83,84,85,86,87,88,89,90,91,92,93,94,95,96,97,98,99,100,101,102,103,104,105,106,107,108,109,110,111,112,113,114,115,116,117,118,119,120,121] articles are described in Supplementary Tables S1 and S2. An overview of the geographical locations of the relevant authors’ institutions shows that the majority of the studies were from Asia (85 studies), followed by America (10 studies) and Europe (1 study), with Chinese and US institutions conducting 87.5% of the studies. A total of 38 studies (39.6%) used cell models, 16 (16.7%) used animal models, and 41 (43.7%) used both (cell + animal) models.

**Figure 2:**
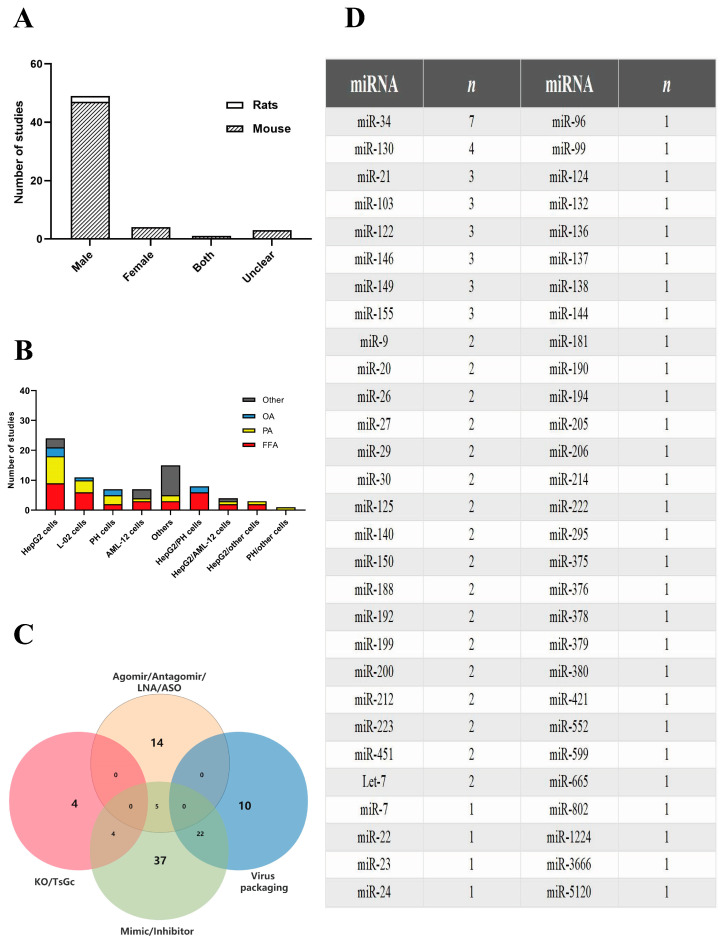
Characteristics of the 96 studies included in this review. Distribution of studies by (**A**) rodent species and sex, (**B**) cell types and induction methods in cell models, (**C**) miRNA delivery methods in cell and animal studies, and (**D**) number of studies for each miRNA variants. AML-12: alpha mouse liver-12, ASO: antisense oligonucleotide, FFA: free-fatty acids, HepG2: human hepatocellular carcinoma, KO: knockout, L-02: hepatocyte 02, LNA: locked nucleic acid, OA; oleic acid, PA: palmitic acid, PH: primary hepatocyte, TsGc: Transgenic mice.

Animal studies were primarily conducted in rodents (96.5% of studies used mice, and 3.5% used rats), with the majority using only males (85.9%) and a minority using only females (7.2%). Overall, 1.7% of the studies used both sexes while 5.2% did not disclose the sex of the animals (Figure 2A). Mouse studies primarily consisted of C57BL/6 (56.1%) or C57BL/6J (35.1%) strains. The mouse models were mainly induced by a high-fat diet (HFD) (71.9%) and a methionine-choline deficient (MCD) diet (12.3%). Animal studies consisted of 85.9% NAFLD and 14.1% NASH models. In the 57 studies using animal models, miRNA agonists/antagonists were mainly administered intravenously (79%) and intraperitoneally (7%), while the remaining studies used miRNA knockout/transgenic animals (14%). The majority of studies intervened NAFLD/NASH with miRNA after the disease onset (68.4%), while the rest (31.6%) introduced miRNA during the induction phase of NAFLD/NASH (including knockout/transgenic mice) (Supplementary Tables S1 and S2).

The NAFLD/NASH cell models mainly used four types of cell lines, namely HepG2 (30%), L-02 (13.8%), PH (8.7%), and AML-12 (8.7%) cells, with some studies (15%) using two cell types in a single study (Figure 2B). NAFLD was induced in the cells using free fatty acids (FFA) mix (PA:OA), palmitic acid (PA), oleic acid (OA), and other mediums such urine acid (UA) and stearic acid (SA). FFA mixture (40.5%) and PA (27.8%) overloads were the most commonly used methods to establish NAFLD/NASH models. Cross-tabulation of cell lines and inducers revealed that HepG2+FFA and HepG2+PA were the most commonly used modeling approaches, and L-02+FFA and L-02+PA were the second most commonly used modeling approaches.

Various forms of miRNA had been used to deliver the nucleic acid materials to the animal and cell models (Figure 2C). miRNAs in the form known as mimics/inhibitors were used as interventions in 37 studies (38.5%). A total of 14 studies (14.6%) used miRNA agomirs/antagomirs/LNA/ASO as interventions, while 5 studies (5.2%) compared two types (mimic/inhibitor vs. agomir/antagomir/LNA/ASO) of interventions. A total of 8 studies (8.3%) used NAFLD/NASH knockout/transgenic models, of which 4 (4.2%) used miRNA knockout models and were treated with miRNA mimics. Besides, 32 studies (33.3%) used miRNAs in the form of viral packaging, and 22 of these studies used miRNA mimics/inhibitors in the form of viral packaging as interventions.

A total of 56 different miRNA variants were used across the 96 studies included in this review, with the majority of studies focusing on miRNA-34 (*n* = 7), followed by miRNA-130 (*n* = 4), miRNA-21 (*n* = 3), miRNA-103 (*n* = 3), miRNA-122 (*n* = 3), miRNA-146 (*n* = 3), miRNA-149 (*n* = 3), and miRNA-155 (*n* = 3) (Figure 2D).

#### 2.1.2. Functional Enrichment Analysis

**Figure 3:**
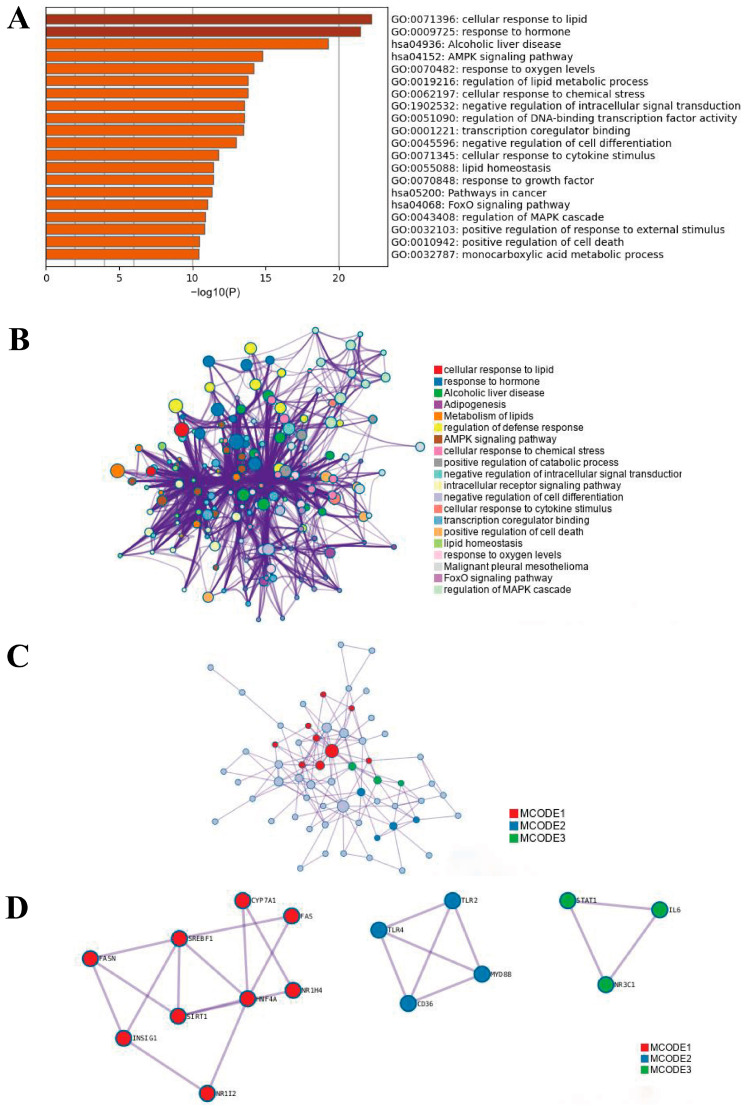
Metascape database analysis of NAFLD/NASH-related pathways targeted by the miRNAs found in this review. (**A**) GO and KEGG enrichment analysis of target signaling pathways associated with the treatment of NAFLD/NASH. (**B**) Network layout of representative terms. The network of enriched terms is colored by cluster-ID. Each terminal is characterized by a circular node whose dimensions are proportional to the number of inputs for that terminal. Nodes with identical cluster-ID are usually in close proximity to each other. (**C**) Network enrichment analysis of GO-biological process for differentially expressed genes (DEGs) regulated by miRNAs. (**D**) Protein-protein interaction (PPI) network diagram of DEGs. MCOD1: Regulation of steroid biosynthetic process, MCOD2: Diseases associated with the Toll-like receptor (TLR) signaling cascade, MCOD3: Response to hormone.

In this review, the target signaling pathways associated with all miRNAs as therapeutic agents for NAFLD/NASH were functionally annotated and enriched using Metascape. The functional enrichment results showed that the pathways were mainly focused on the representative terms of cellular response to lipids, hormone response, alcoholic liver disease, and the AMPK signaling pathway (Figure 3A,B). By mapping the protein-protein interaction (PPI) network of differentially expressed genes (DEGs) regulated by these miRNAs, three gene clusters in the PPI network were mapped to 16 target DEGs such as silencing information regulatory factor 2-related enzyme 1 gene (SIRT1), fatty acid synthase gene (FASN), sterol regulatory element binding transcription factor 1 gene (SREBF1), and other genes that regulate the pathway of lipid and glucose metabolism in liver cells (Figure 3C,D). In addition, the target genes also include CD36 and Toll-like receptor 1/2 gene (TLR1/TLR2) which regulate innate immunity and inflammation-promoting mechanisms in hepatocytes. The third gene cluster is involved in the signal transducer and activator of transcription 1 gene (STAT1), interleukin (IL6) and nuclear receptor subfamily 3C group member 1 gene (NR3C1), which regulate hepatocyte proliferation and adaptive immune response.

#### 2.1.3. Biologic Processes

**Figure 4:**
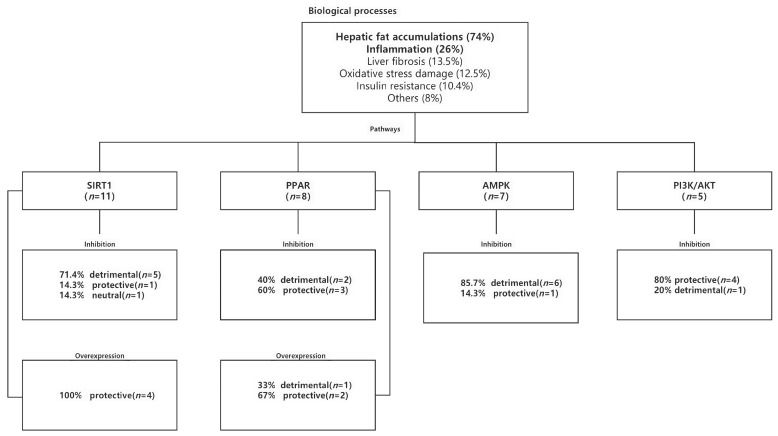
The primary biological processes and signaling pathways as reported to be modulated by the miRNAs included in this review. Hepatic fat accumulation and inflammation were the predominant biological processes involved in the miRNA interventions while SIRT1 was the main targeted pathway. Inhibition or overexpression of the genes in the pathways by miRNAs may result in protective (therapeutic), detrimental, or neutral effects towards NAFLD/NASH. AMPK: adenosine 5′-monophosphate (AMP)-activated protein kinase 3, AKT: protein kinase, PI3K: phosphatidylinositide 3 kinase, PPAR: peroxisome proliferator-activated receptor, SIRT1: sirtuin 1.

This review found 14 potential biological processes that were reported to be modulated by the miRNAs in NAFLD/NASH from the 96 included studies (Supplementary Table S2 and Figure 4). These processes included hepatic fat accumulation (*n* = 74, 77%), inflammation (*n* = 25, 26%), liver fibrosis (*n* = 13, 13.5%), oxidative stress damage (*n* = 12, 12.5%), insulin resistance (*n* = 10, 10.4%), and others (*n* = 8, 8%). Both hepatic fat accumulation and inflammation were found to be modulated together by miRNA in 15.6% (*n* = 15) of studies.

SIRT1 was the most reported signaling pathway in all 96 studies (*n* = 11, 11.5%) (Figure 4). All four miRNA intervention studies that led to overexpression of SIRT1 found a protective (therapeutic) effect against NAFLD/NASH. Concomitantly, five of the seven studies that inhibited SIRT1 expression after the application of miRNAs reported an opposing (detrimental) effect on NAFLD/NASH. Three of the seven studies using miRNA 34 reported experimental validation of SIRT1 as a mechanistic signaling pathway involved in fat accumulation, while the remaining four studies showed the regulatory role of SIRT1 in lipid metabolism (Supplementary Table S1).

Eight studies noted the involvement of the peroxisome proliferator-activated receptor (PPAR) signaling pathway in miRNA-based interventions. In three miRNA studies that resulted in overexpression of PPAR, two studies reported a protective effect against NAFLD/NASH (Supplementary Table S1 and Figure 4). However, the findings were not consistent with other studies that reported the inhibition of PPAR on NAFLD/NASH. When PPAR expression was inhibited following miRNA intervention, only two out of five studies reported an opposing (detrimental) effect against NAFLD/NASH. These findings indicate that the role of PPAR in mediating the therapeutic effect of miRNAs remains complex.

Among the seven studies that reported on the involvement of the AMPK signaling pathway, six studies found that inhibition of the pathway following miRNA interventions resulted in deleterious effects (Supplementary Table S1 and Figure 4). Among the five studies that examined the effect of miRNA interventions on the PI3K/AKT signaling pathway, four studies noted that inhibition of the pathway resulted in a protective effect against NAFLD/NASH (Supplementary Table S1 and Figure 4).

#### 2.1.4. Meta-Analysis of miRNA Interventions on NAFLD/NASH

**Figure 5:**
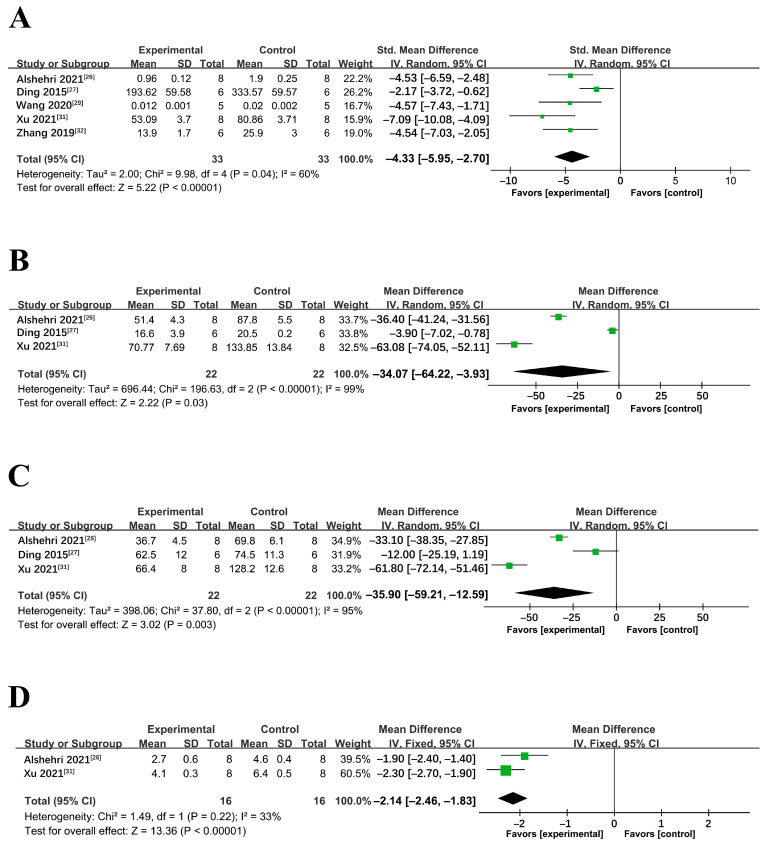
Meta-analyses of miRNA-34a antagonist on (**A**) hepatic TG, (**B**) alanine aminotransferase, (**C**) aspartate aminotransferase, and (**D**) hepatic TC in NAFLD/NASH models (in the presence or absence of pre-conditioning). TC: Total Cholesterol, TG: Triglycerides, SD: standard deviation, CI: confidence interval.

Of the seven studies that determined the role of miRNA-34a in NAFLD/NASH animal models, five involved miRNA-34a antagonists and two used locked nucleic acids (LNA) inhibitor targeting miRNA-34 [26,27,28,29,30,31,32]. Hepatic triglyceride (TG) levels were reported in five studies, serum alanine aminotransferase (ALT) and serum aspartate aminotransferase (AST) in three studies, and hepatic total cholesterol (TC), serum TG, and serum TC in two studies. These studies were combined for meta-analysis regardless of variations in research design, NAFLD/NASH models, or behavioral characteristics. Pretreatment with miRNA-34a antagonist significantly reduced hepatic TG levels (mean difference: 4.33 mg/g; 95% CI: 2.70, 5.95; *p* < 0.00001), despite the high heterogeneity of the studies (*p* = 0.04) (Figure 5A). In addition, miRNA-34a antagonist significantly reduced ALT (mean difference: 34.07 U/L; 95% CI: 3.93, 64.22; *p* = 0.03) (Figure 5B) and AST (mean difference: 35.90 U/L; 95% CI: 12.59, 59.21; *p* = 0.003) although there was high heterogeneity (*p* < 0.00001) (Figure 5C). miRNA-34a antagonist also significantly reduced hepatic TC levels (mean difference: 2.14 mg/g; 95% CI: 1.83, 2.46; *p* < 0.00001) with low heterogeneity between studies (*p* = 0.22) (Figure 5D).

**Figure 6:**
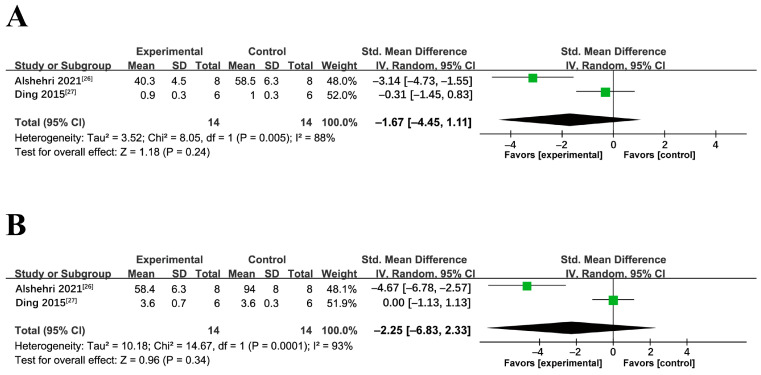
Meta-analyses of miRNA-34a antagonist on serum (**A**) TG and (**B**) TC in NAFLD/NASH models (in the presence or absence of pre-conditioning). TC: Total Cholesterol, TG: Triglycerides, SD: standard deviation, CI: confidence interval.

The effect of miRNA-34a inhibition on serum TG was not apparent (*p* = 0.24) with high heterogeneity (*p* = 0.005) (Figure 6A). Similarly, no overall effect was observed on serum TC after miRNA-34a inhibition (*p* = 0.34) with significant heterogeneity (*p* = 0.0001) (Figure 6B).

Notably, the review on the antagonistic effects of miRNA-34a identified hepatic fat accumulation as the primary biological process involved (Supplementary Table S2). Only two studies explored the role of miRNA-34a inhibition in reducing inflammatory responses and fibrosis as well as in improving insulin resistance in NAFLD/NASH models.

Meta-analyses for other miRNAs such as miRNA-130, miRNA-21, miRNA-103, miRNA-122, miRNA-146, miRNA-149, and miRNA-155 were hampered by insufficient data and a high degree of statistical heterogeneity between studies.

#### 2.1.5. Quality Assessment

**Figure 7:**
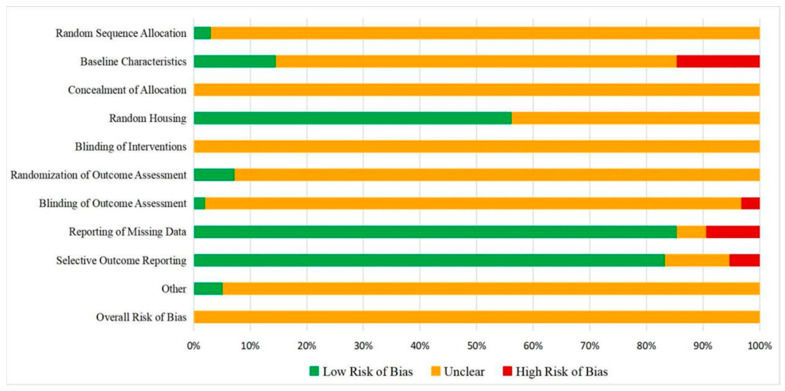
Risk of bias analysis was performed on the 96 included research studies with the SYRCLE ROB tool. The low risk of bias is presented in green, the unclear risk is presented in orange, and the high risk of bias is presented in red.

All 96 included studies were assessed using the SYRCLE ROB tool. Results showed that all studies had unclear risk assessment because they lacked clear descriptions of baseline characteristics (88.17%), blinding (of outcome evaluators) (96.8%), data reporting (90.6%), and study protocol (94.7%). Information on animal weights, method of randomization, sample size calculation, and study protocols were generally not reported in detail in the studies (Figure 7). Only the data from studies involving miRNA-34a were found to be of medium quality.

#### 2.1.6. Publication Bias

Statistical tests for asymmetry of scatter plots and reporting bias were not performed due to the uncertainty of bias risk of most studies, high statistical heterogeneity (>90%), and the limited number of studies (<10).

### 2.2. Discussion

Despite the apparent heterogeneity in the design characteristics of most of the studies in this systematic review, several significant findings are worth emphasizing despite the apparent heterogeneity in design characteristics for most studies. All investigations reported remarkable impacts (protective or detrimental) of miRNA interventions on NAFLD/NASH progression, mainly in lipid metabolism and inflammatory responses. In addition, the studies collectively emphasized the involvement of miRNAs in the pathogenesis and therapeutic application of NAFLD/NASH.

miRNA sequences are strongly conserved across species and have a similar tissue distribution in animals (rodents and rabbits) and humans [115]. A large number of miRNAs are expressed in mammalian liver and influence disease development by altering RNA expression patterns (e.g., overexpression or repression) in disease states [116]. The biological effects of miRNAs are complex and varied because their modes of action include classical and non-classical pathways. The classical pathway first generates pri-miRNA transcripts while the non-classical pathway produces small hairpin RNA (shRNA), mirtron, or 7-methylguanine capped-pre-miRNA in the nucleus. These primary miRNAs are further cleaved by Drosha and DiGeorge Syndrome Critical Region 8 (DGCR8) to produce pre-miRNA. The pre-miRNA transcripts are transported to the cytoplasm, where they are further processed to produce mature miRNA which forms a miRNA-induced silencing complex (miRISC). The miRISC binds to the target mRNA and represses translation by degrading the target mRNA [115].

There are currently two types of miRNA-based therapeutic strategies: replacement, restoration, or overexpression therapy, i.e., miRNA replacement/overexpression therapy, and reduction, inhibition, or down-regulation therapy, i.e., miRNA reduction/inhibition therapy. miRNA replacement/overexpression therapies restore/enhance endogenous miRNAs mainly through chemically synthesized miRNAs or miRNA mimics. These synthetic miRNAs have the same sequences and indistinguishable biological roles as mature endogenous miRNAs. They can be transported to the cytoplasm via different transporters, such as reagents or virus packaging. These synthetic miRNAs are linked to RISC in the cytoplasm [115]. On the other hand, miRNA reduction/suppression therapies inactivate disease-causing miRNAs that are downregulated in disease by different antagonists that possess a complementary sequence to the targeted endogenous mRNA. These methods include miRNA inhibitors and oligomers. miRNA inhibitors are also transferred into the cytoplasm by different transporters, such as reagents or virus packaging. Antisense oligonucleotide (ASO), locked nucleic acid (LNA) are the most common antisense nucleotide species. Knockouts or transgenic mice can also be used in preclinical studies to reduce the expression of endogenous miRNAs. However, the most significant disadvantage of this approach is that it is not suitable for clinical application [115].

In the present review, we included 96 studies that investigated the therapeutic effects of a total of 56 different miRNA variants on NAFLD/NASH. These miRNAs were mainly involved in hepatic lipid accumulation, inflammation, and liver fibrosis of NAFLD/NASH models. The SIRT1 signaling pathway is the most common target by these miRNAs such that overexpression of SIRT1 by miRNA interventions has been shown to have a protective effect against NAFLD/NASH. The signaling events involving miRNA intervention in NAFLD/NASH may be a result of the binding of different miRNAs to multiple target genes in the signaling pathways that lead to a common signaling event.

miRNA-34 is highly evolutionarily conserved in mammals, with only two distinct gene coding sequences in the miRNA-34 family. The miRNA-34a sequence is located on chromosome 1p36.23 while the miRNA-34b and miRNA-34c sequences are located together in a primitive transcriptional cluster on chromosome 11q23.1 [117]. miRNA-34a is expressed in the pancreas, liver, and heart. Previous research has also demonstrated that miRNA-34a is found to be expressed at elevated levels in drug-induced adipocytes, in the construction of NAFLD/NASH mouse models, and in the liver cells of patients.

miRNA-34a antagonist has a significant protective effect on NAFLD/NASH models presented with bile acid abnormalities and metabolic syndrome preadaptation [117,119]. Wen et al. [119] found that fat accumulation could alter cellular mitochondrial morphology and lead to abnormal cellular mitochondrial function. miRNA-34a antagonists significantly restored the transmembrane potential of cellular mitochondria, improved cellular mitochondrial function, and reduced fat accumulation. Thounaojam et al. [120] showed that a high glucose diet reduced histone deacetylase SIRT1, which is associated with reduced cellular mitochondrial function and loss of mitochondrial biogenesis factors (i.e., PGC-1α, NRF1, and TFAM). Transfection of miRNA-34a antagonist into cells prevented high glucose-induced cellular mitochondrial dysfunction and upregulation of cellular senescence-related markers.

miRNA-34a mimics promoted mitochondrial dysfunction and cellular senescence, presumably through the inhibition of the SIRT1-PGC-1α-NRF1 pathway. Other studies [121,122] found that fat accumulation, insulin resistance, and liver damage in NAFLD mouse models were improved after treatment with green tea and gynostemium, while further studies on liver tissues revealed a significant decrease in miRNA-34a. This result suggested that green tea and gynostemium altered liver metabolism through epigenetic regulation of miRNA-34a. Another study [123] found circRNA-0046367 to be an endogenous regulator of miRNA-34a. circRNA-0046367 was significantly downregulated in liver tissues of patients with NAFLD while miRNA-34a levels were elevated. The downregulation of circRNA-0046367 promoted the inhibitory effect of miRNA-34a on peroxisome proliferator-activated receptor alpha (PPARα) and exacerbated the development of steatosis.

Wu et al. [124] suggested that sterol regulatory element binding protein 1c (SREBP-1c) regulates lipid homeostasis through the activation of several fatty acid synthesis-related enzymes. Overexpression of miRNA-34a significantly upregulates SREBP-1c levels through SIRT1 and thus leads to lipid metabolism disorders. Wu et al. [125] showed that upregulation of miRNA-34a in patients with NAFLD led to inhibition of SIRT1, and inhibition of miRNA-34a promoted SIRT1 synthesis, allowing activation of PPARα and AMPK. In addition, activation of PPARα and AMPK stimulated lipolysis and metabolism. Min et al. [126] showed that miRNA-34a mimic suppressed SIRT1 expression by activating AMPK and dephosphorylating hydroxymethylglutaryl-coenzyme A reductase (HMGCR).

Another study [127] reported that Creb-regulated transcriptional coactivator (Crtc)2 is another primary regulator of systemic energy metabolism that involves miRNA-34a in its mechanism of action. In the Crtc2-specific knockout mouse model of NAFLD, plasma and liver fibroblast growth factor 21 (FGF21) levels were found to be elevated. The degree of oxidative stress in the mouse liver cells was alleviated and expression of miR-34a was downregulated, due to high expression of SIRT1 and PPARα. However, ectopic overexpression of miRNA-34a was followed by suppression of Sirt1 and PPARα expression, along with increased oxidative stress in mouse liver cells. Therefore, CREB/Crtc2 was suggested to induce miRNA-34a to negatively regulate the Sirt1/PPARα/FGF21 axis.

We reviewed seven studies addressing the therapeutic efficacy of miRNA-34a in NAFLD/NASH animal models, which collectively agreed that miRNA-34a plays a role in disease progression [26,27,28,29,30,31,32]. In the present meta-analysis, in which miR-34a antagonists were used as an intervention, hepatic TG, TC, ALT, and AST levels were significantly reduced, supporting the hypothesis that endogenous miRNA-34a is detrimental to NAFLD/NASH. Indeed, the high expression of miRNA-34a in the liver induced by HFD may be related to its pathogenic role in NAFLD/NASH. Inhibition of miRNA-34a reduced intracytoplasmic lipid droplet deposition in most studies. These studies [26,27,28,29,30,31,32] explored the possibility of SIRT1 as a miRNA-34a target, with one-third of these studies [26,27,29] identifying SIRT1 as a critical target of miRNA34a. Inhibition of miRNA-34a increased hepatic SIRT1 expression, activated fatty acid oxidation, and inhibited hepatocyte steatosis. It was also found that inhibition of hepatic miRNA-34a reduced hepatocyte ROS levels and decreased gene expression of fibrogenesis (TGFβ, α-SMA and TIMP1). Therefore, miRNA-34a may be involved in the progression of NAFLD to NASH by promoting lipid uptake and synthesis, inducing inflammation, ROS activation, and apoptosis. However, the effect of miRNA-34a mimicry or overexpression in NAFLD/NASH has yet to be reported. Thus, meta-analysis was restricted to miRNA-34a antagonistic studies. Further studies should investigate the protective effects of this miRNA antagonist at different phases of the disease and the dosage needed to establish therapeutic efficacy in human trials.

Wang et al. [22] used miRNA microarray analysis and found that miRNA-130a-3p was remarkably downregulated in the liver of NAFLD mice undergoing liver fibrosis, which was further confirmed in liver tissues of MCD-fed mice and patients with NASH. miRNA-130a-3p mimics were transfected into HSC-T6 cells, which in turn directly acted on TGFBR1 and TGFBR2 through the TGF-β/Smad signaling pathway, thereby inhibiting the activation and proliferation of hepatic stellate cells (HSCs). Through bioinformatics analysis and dual luciferase assay, Liu et al. [24] verified that miRNA-130a bound directly to LncRNA-H19 and that overexpression of LncRNA-H19 promoted TG secretion and lipid accumulation in hepatocytes by downregulating miRNA-130a to activate the PPARγ pathway. Knockdown of LncRNA-H19 or overexpression of miRNA-130a reversed this change.

Using the Starbase database, Guo et al. [25] confirmed that HOTAIR binds to miRNA-130b-3p. The effect of miRNA-130b-3p on its target ROCK1 was verified by dual luciferase assay in FFA-induced HepG2 cells. Further studies showed that activation of ROCK1 increased fat accumulation. In the hepatic tissue of HFD-fed NAFLD mice, significant increases in levels of HOTAIR and ROCK1 were observed, and the expression of miRNA-130b-3p was suppressed, thus confirming that HOTAIR can promote NAFLD through the miRNA-130b-3p/ROCK1 axis. However, Liu et al. [23] found that hepatic fat accumulation and insulin resistance were significantly improved in HFD-fed knockout mice of miRNA-130b-5p. Similar changes were achieved first by artificial downregulation of miRNA-130b-5p and then further upregulation of insulin-like growth factor binding protein 2 (IGFBP2). However, there was no improvement in hepatic lipid accumulation and insulin resistance in mice with simultaneous downregulation of miRNA-130b-5p and IGFBP2. The expression of IGFBP2 and the degree of AKT/AKT phosphorylation were synchronous, i.e., enhanced or reduced simultaneously. Thus, miRNA-130b-5p downregulation in the NAFLD model could improve lipid accumulation and insulin resistance in NAFLD mice by activating the AKT pathway in an IGFBP2-dependent manner.

As reported in this review, studies that used miRNA-130 mimics have supported the protective properties of this miRNA in NAFLD/NASH. However, studies with miRNA-130 knockout mice have yielded an opposite finding (i.e., protective effect after downregulation of miRNA-130) [22,23,24,25]. Differences in the precursor and stem-loop arms [128] of miRNA-130 used in these studies, as well as a limited number of studies (*n* < 2), resulted in increased heterogeneity, which hindered further meta-analysis. Based on previous findings, miRNAs are derived from either the 5p or 3p arm, which show dynamic expression and evolutionary patterns. The arm selection and/or arm switching control miRNA expression and functionality in response to biological needs [128]. Hence, more preclinical studies are necessary to investigate the role of precursors and stem-loop arms of miRNA-130 in NAFLD/NASH.

Clinical data has shown that high miRNA-21 expression is associated with poor clinical prognosis of NAFLD. miRNA-21 is significantly elevated in the livers of patients with NASH [129,130]. Several in vitro and in vivo studies have consistently demonstrated that miRNA-21 can regulate the expression of crucial metabolic transcription factors such as insulin sensitivity, fatty acid uptake, and lipid synthesis [64,65,66]. Research [66] has demonstrated that miRNA-21 is upregulated in the hepatic tissue of patients with NAFLD/NASH, and this was confirmed in HFD-fed mice and fatty acid-intervened cells.

Bioinformatics of the HBP1 mRNA demonstrated that the 3′-UTR of HBP1 mRNA was 100% in complementarity with the miRNA-21 and that LNA anti-miRNA-21 transfection of OA-treated HepG2 cells resulted in a significant increase in HBP1 and a subsequent decrease in fat accumulation. Supplementation with HBP1-siRNA counteracted the effect of LNA anti-miRNA-21. In further animal studies, mice administered with LNA anti-miRNA-21 demonstrated an increase in HBP1 and a significant decrease in hepatocyte TG levels, leading to the combined inference that miRNA-21 played an essential part in NAFLD by its interactions with the HBP1 pathway. Rodrigues et al. [64] showed a significant increase in PPARα protein levels after administration of a methionine and choline-deficient diet (MCD) to miRNA-21 knockout mice, but no progressive increase in TNF-α, IL-1β, serum ALT, and caspase-2 levels; additionally, they found a noticeable reduction in hepatic steatosis. miRNA-21 ablation resulted in a progressive reduction in fat accumulation, inflammation, and apoptosis in hepatocytes of NAFLD/NASH mice, and slowed the onset of fibrosis.

Wang et al. [65] injected miRNA-21 antagonists into MCD-fed NAFLD mice, and the levels of serum lipids (TG, TC, LDL) and transaminases (ALT, AST) were decreased. The expression of lipid synthesis-related genes (SREBP1c and FASN) was inhibited, confirming that fat accumulation and inflammation associated with NAFLD progression to NASH can be reduced by inhibition of miRNA-21. Notably, all three studies [64,65,66] involving miRNA-21 supported the protective effect of inhibiting this miRNA in NAFLD/NASH models, which suggests miRNA-21 antagonist as a potential therapeutic target for treating NAFLD/NASH and metabolic syndrome. However, a meta-analysis could not be conducted due to differences in the observed indicators and the limited number of observed indicators across studies (*n* < 2).

Recent studies reported that miRNA-146 reduced hepatic lipid accumulation and NASH in NAFLD mice [131,132]. He et al. [74] found significantly reduced levels of miRNA-146b in the livers of MCD-fed mice. They also found that miRNA-146b deletion promoted liver inflammation and fibrosis in MCD-fed knockout miRNA-146b mice. The miRNA-146b mimics were injected into mice by nanoparticle technology and revealed a significantly reduced inflammation and lipid droplet formation in mouse hepatocytes, along with a significant decrease in serum ALT and AST levels, which suggests that miRNAs nanoparticles can be explored as a therapeutic agent for NAFLD treatment. Chen et al. [73]. applied bioinformatic characterization and identified potential miRNA-146a-5p binding sites for NEAT1 and the 3′ UTR of ROCK1 through bioinformatics. FFA-induced dual luciferase in HepG2 cells showed that NEAT1 restrained the expression of miR-146a-5p and promoted the performance of ROCK1. Further experiments showed that transfection of miRNA-146a-5p mimics significantly decreased ROCK1, TG, and lipid accumulation while transfection with pcDNA-Rock1 disrupted the inhibitory effect of miRNA-146a-5p mimics. Transfection of sh-NEAT1 reversed the excessive lipid accumulation caused by FFA, but the reversal effect of sh-NEAT1 was inhibited when Sh-NEAT1 acted simultaneously with miR-146a-5p inhibitor. Transfection with sh-ROCK1 also attenuated FFA-induced lipid formation, thus confirming that NEAT1 can promote NAFLD by inhibiting miRNA-146a-5p, upregulating ROCK1, and promoting hepatic lipid accumulation. The mammalian-mediated complex MED1, identified by Li et al. [75] through Targetscan database analysis, is a target gene for miRNA-146a. miRNA-146a expression levels were significantly reduced in ob/ob mice, HFD-fed NAFLD mice, and FFA-induced AML-12 cells. Serum insulin levels and liver TG were found to be significantly reduced in mice injected with miRNA-146a mimics. The negative effects of the miRNA-146a inhibitor on the mitochondrial function and lipid metabolism of FFA-induced AML-12 cells were enhanced after overexpression of MED1 using pcDNA3-MED1. The effect of miRNA-146a on mitochondrial function and glycolipid metabolism were found to be mediated by MED1. miRNA-146 overexpression treatment significantly improved the occurrence of fat accumulation in hepatocytes. Thus, intervention with miRNA-146 mimics might represent a promising new direction for the treatment of NAFLD to NASH.

Zhang et al. [69]. found miRNA-103 to be highly complementary to the 3′-UTR of FASN mRNA through the TargetScan genetic database. Dual luciferase reaction strongly suggested that direct miRNA-103 binding to the 3′-UTR of FASN mRNA was required for FASN inhibition. Overexpression of miRNA-103 in high-cholesterol diet (HCD)-fed NAFLD mice and genetically obese (db/db) mice resulted in a significant reduction in FASN, reduced liver weight, and TG in mice. In addition, Chu et al. [67] applied the GSE65978 microarray from the GEO database to analyze and select the first 30 significantly differentially expressed miRNAs and validated them with serum from patients with NAFLD and liver tissues of HFD-fed NAFLD mice to confirm the upregulated miRNA-103a-3p expression in these samples. Nevertheless, in further animal tests, when mice were given miRNA-103a-3p inhibitors, this increased HBP1 and significantly decreased the serum TG, TC, ALT, and AST levels of the mice. Further injection of sh-HBP1 into NAFLD mice treated with miR-103a-3p inhibitors increased hepatic fat accumulation, inflammation, and fibrosis, and attenuated the impact of miR-103a-3p inhibitors in the mice. It is, therefore, inferred that miRNA-103a-3p plays an essential role in NAFLD by its interaction through the HBP1 pathway. Furthermore, Ding et al. [68] found increased expression of miRNA-103-3p both in FFA-treated L02 cells and in hepatocytes of NAFLD mice. The administration of miRNA-103-3p antagonists reduced lipid droplet aggregation in L02 cells and significantly reduced inflammatory responses, abnormal lipid metabolism, and oxidative stress in NAFLD mice. Therefore, inhibition of miRNA-103-3p offers a potential therapeutic strategy for treating NAFLD. The contradictory findings may be attributed to different forms of mature miRNA sequences produced in these disease models [128], and so, the therapeutic role of miRNA-103 for NAFLD still needs further evaluation at the structural level of the miRNA itself.

miRNA-122 is one of the most abundant miRNAs in the liver and is essential in regulating lipid metabolism. The levels of miRNA-122 are positively correlated with disease severity [130,133]. In human hepatic stellate cells (HSCs), the production of pro-inflammatory cytokines was inhibited by miR-122 through the targeting of the PKR-activating protein (PACT) [134]. Long et al. [72] found elevated miRNA-122 expression in both the hepatic cells of HFD-fed NAFLD mice and FFA-induced HepG2 and Huh-7 liver cells. miRNA-122 action on the direct target SIRT1 was verified by dual luciferase assay. miRNA-122 inhibitor action on the NAFLD cell model inhibited FFA-induced TG secretion, while silencing SIRT1 reversed this phenotype, thus confirming that miR-122 inhibitors protected hepatocytes from lipid deposition and inhibited the development of NAFLD by promoting the expression of SIRT1. Hu et al. [71] identified significantly increased levels of miRNA-122-5p both in HFD-fed NAFLD mice as well as in PA-induced L02 cells. FOXO3 was further demonstrated to be a potential target of miRNA-122-5p by a dual luciferase assay. miRNA-122-5p inhibitors reduced hepatic TG, hepatic TC, and inflammatory cytokines (TNF-α, IL-6, and IL-8), and administration of siFOXO3 reversed the exacerbation of fat accumulation and immune cell infiltration. Thus, inhibition of miRNA-122-5p/FOXO3 axis could be a target for the potential therapy of NAFLD. However, a study by Chai et al. [70] comparing miRNA in the liver of patients with NASH with that of healthy individuals found that miRNA-122 was reduced in the patients. Furthermore, analysis of the human NASH dataset found that miRNA-122 was negatively correlated with RORA. Injections of miRNA-122 inhibitors in HFD-fed NASH mice revealed that reduced miRNA-122 was associated with increased hepatic TG and muscle TG levels, while further injection of RORA agonist (RS-2982) reduced fat accumulation, inflammation, and reverse fibrosis in NASH mice by increasing miRNA-122 expression. These discrepancies could be due to the disease stages where inhibition of miRNA-122 was therapeutic for NAFLD while its inhibition was detrimental in NASH, though the reasons remain to be explored. The role of miR-122 in hepatic stellate cells (HSCs) may influence hepatic inflammation and fibrosis as shown by a previous study [134].

Xiao et al. [78] demonstrated a significantly elevated expression of miRNA-149 in hepatic tissues of both FFA-induced HepG2 cells and NAFLD mice treated with HFD. miRNA-149 promoted intracellular adipogenesis in the absence of FFA induction, but it did not promote further intracellular adipogenesis in the absence of FFA induction. In contrast, inhibition of miRNA-149 in the presence of FFA reduced adipogenesis in hepatocytes. According to the miRWalk database, FGF-21 is a miRNA-149 target gene, and FGF-21 siRNA blocks the adipogenesis-inducing inhibitory effect of miR-149 inhibitors on FFA-induced adipogenesis in HepG2 cells. Thus, inhibition of miRNA-149 might become a promising new therapeutic for the treatment of NAFLD. Through gene microarray and animal experiments, Chen et al. [76] also demonstrated that miRNA-149-5p was dramatically upregulated in mice fed with HFD. Overexpression of miRNA-149-5p significantly enhanced UA-induced TG accumulation in HepG2 cells, and inhibition of miRNA-149-5p improved TG accumulation. FGF21 was demonstrated to be a gene target of miRNA-149-5p through luciferase assay, and silencing FGF21 eliminated the ameliorative impact of miRNA-149-5p inhibitors on lipid accumulation. In contrast, overexpression of FGF21 prevented miRNA-149-5p mimics-induced lipid accumulation in hepatocytes. However, Chen et al. [77] demonstrated that miRNA-149 expression was substantially less in NAFLD mice fed with HFD than in normal mice. In comparison, the mRNA and protein expression associated with the ATF6 signaling pathway was substantially higher in NAFLD than in normal mice. miRNA-149 mimics transfected with NAFLD mouse hepatocytes showed significantly lower mRNA and protein expression of the ATF6 signaling pathway and lower expression of ERS-related factors (XBP1 and GRP78). miRNA-149 negatively regulates the ERS-induced inflammatory response and apoptosis in NAFLD through the ATF6 signaling pathway and restrains the ongoing progression of NAFLD. In miRNA-149 research, two studies [76,78] concluded that inhibition of miRNA-149 reduced hepatocyte adipogenesis and fat accumulation, and one concluded [77] that the overexpression of miRNA-149 reduced the inflammatory response and apoptosis in NAFLD. These contradictory findings suggest the existence of multi gene targets for miRNA-149 which could be attributed to the fact that the expression of miRNA depends on 5p or 3p arm selection of the pre-miRNA during adaption to functional and environmental stressors [128]. Therefore, a comprehensive analysis, and further experimental studies at the miRNA level, are necessary to determine the therapeutic efficacy of miRNA-149 for NAFLD.

Cask et al. [80] discovered highly expressed miRNA-155 in the hepatocytes of MCD-fed NASH mice. Hepatic fat accumulation and fibrosis were improved in mice knocked out of miRNA-155, but there was no significant attenuating effect on steatohepatitis. Bala et al. [79] discovered that the hepatic levels of miRNA-155 were significantly increased in patients with NASH. After miRNA-155 knockout mice were given a high-fat, high-cholesterol, high-sugar (HF-HC-HS) diet, the mice’s liver damage, inflammatory response, and fibrosis were significantly reduced compared to normal mice. miRNA-155 can target various genes that bind to critical cellular events in NAFLD/NASH (e.g., fat accumulation, inflammation, fibrosis, etc.). However, Wang et al. [81] identified a significant reduction in miRNA-155 in hepatic and peripersonal blood specimens from patients with NASH. miRNA-155 overexpression reduced lipid accumulation in FFA mixture-induced Hep1-6 cells, and upregulation of miRNA-155 reduced TG content in hepatocytes from HFD-fed NAFLD mice. Furthermore, overexpression of miRNA-155 in C57BL/6J mice with NAFLD reduced hepatic TG content. The contradictory findings might be due to the base inconsistencies in different mature sequences of the miRNA-155 which result in differential therapeutic efficacy for NAFLD [128]. The therapeutic efficacy of miRNA-155 for NAFLD still needs further validation.

The beneficial effects of miRNA intervention were most often associated with improved lipid metabolism, reduced inflammatory responses, and activation of the SIRT1 signaling pathway. However, the limited number of studies and heterogeneity of study designs reduced the overall intensity of the evidence. Despite the promising therapeutic effects of miRNAs, there is still a scarcity of evidence to conclude the efficacy of an individual miRNA treatment in NAFLD/NASH. There is an apparent risk of biases in most of the studies, partly due to the limited description of baseline characteristics, methodology, and data reporting in the publications. The animal models were restricted to population diversity, as they were conducted only in rodents and mostly in males. The lack of reported adverse effects following miRNA intervention or effects on other organ functions besides the liver in the selected studies showed the inadequacy of the preclinical trial designs. Because of these limitations, the significance of preclinical data for miRNA applications in human disease is still inconclusive. Only a few studies have described the co-morbidities involved in NAFLD/NASH development such as diabetes and metabolic syndrome.

While challenges remain in the translation of these preclinical findings into humans for the treatment of NAFLD/NASH, it is interesting to note that human clinical trials involving miRNAs as therapeutics in other diseases have been accomplished or are in the development pipeline. For example, Miravirsen, a newly developed drug, has been used to inhibit the expression of miR-122 in the treatment of hepatitis C virus (HCV) infection in humans. The phase 2 clinical trial of this drug showed that patients did not develop liver cancer or other liver-related complications [135,136]. Therefore, miRNAs are potential therapeutic targets for the treatment of liver diseases. More carefully designed preclinical studies involving miRNA therapies in NAFLD/NASH that limits biases and ensures safety checks are essential for the advancement of miRNA therapy in patients with this disease.

## 3. Materials and Methods

The reported quality of the included studies was assessed according to the Systematic Evaluation and Meta-Analysis (PRISMA) statement to explore the therapeutic effects of miRNAs on NAFLD/NASH. Searches were restricted to articles written in English. PubMed, Cochrane, and Scopus databases were used by entering the MeSH subject: NAFLD, fatty liver, steatosis, and combinations of these MeSH terms. A manual search of all original articles, conference reports, abstracts, and posters was conducted to collect all available data until 2 February 2023. Inclusion criteria were defined as (1) studies using cells or animals in NAFLD and NASH models; (2) inhibition or overexpression of a single miRNA by one or more measures; (3) existence of controlled experiments; (4) publications written in English. Exclusion criteria were (1) more than one miRNA intervention in a study; (2) lack of assessment on therapeutic effect; (3) experimental studies without negative control; (4) non-English publication; (5) no relevant outcomes reported.

Three investigators (Y.Z.Z., J.A.G. and J.K.T.) independently screened the titles and abstracts of each study. In cases of disagreement about the selected studies, meetings were arranged for consultation to resolve potential differences through open discussion. Details were as follows: (1) study characteristics: author, publication, year, study site (Country/Region); (2) animal models: species, sex; (3) intervention and control characteristics: miRNA variants, dose, timing of administration, route of administration; (4) intervention outcomes: fat accumulation, inflammation, hepatocellular fibrosis, oxidative stress, insulin resistance, and others.

The functional enrichment analysis was performed using the Metascape database (https://metascape.org/; accessed on 12 January 2023). The meta-analysis was conducted using RevMan 5.4.

## 4. Conclusions

**Figure 8:**
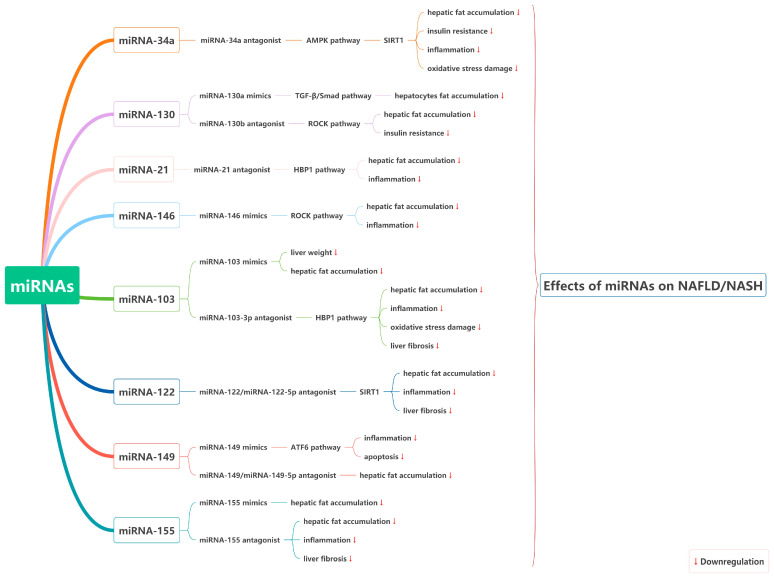
The therapeutic targets and effects of miRNAs in NAFLD/NASH.

In conclusion, due to the high prevalence and therapeutic complexity of NALFD/NASH, the application of miRNA-based therapeutic approaches in the treatment of NAFLD/NASH seems attractive because miRNAs are pleiotropic molecules that can regulate multiple dysregulated genes and/or signaling pathways simultaneously. However, the pleiotropic role of miRNAs can be a double-edged sword, because until all possible molecular targets of each miRNA are fully understood, their inhibition or overexpression for therapeutic purposes will not be fully controlled from a clinical treatment perspective. The application of miRNA therapeutics is still an immature field, despite the fact that numerous scientific teams and companies have invested significant efforts in the development of miRNA-based drugs. The potential therapeutic targets and effects of miRNAs in NAFLD/NASH are illustrated in Figure 8. Although the studies in this review for miRNA-34a inhibitors, miRNA-21 inhibitors, and miRNA-146 agonists consistently show positive therapeutic effects in NAFLD/NASH, none go beyond preclinical studies. The main direction for the future development of miRNA therapy is to conduct more in-depth studies on the targets of these molecules to provide a more comprehensive and in-depth overview of the signaling pathways that miRNAs can regulate. A comprehensive understanding of the targets of miRNAs and a more precise selection of inhibitors or agonists is more in line with the contemporary concept of precision medicine.

## Data Availability

Data available on request.

## Supplementary Materials

The following supporting information can be downloaded at: https://www.mdpi.com/article/10.3390/ijms24119168/s1.
