# Supplementary material for: Therapeutic Effects of microRNAs on Nonalcoholic Fatty Liver Disease (NAFLD) and Nonalcoholic Steatohepatitis (NASH): A Systematic Review and Meta-Analysis
Source: Int J Mol Sci. 2023 May 23;24(11):9168. doi: 10.3390/ijms24119168 (PMC10252239; doi:10.3390/ijms24119168)
Supplement: Supplementary file 1 [file ijms-24-09168-s001.zip › Supplementary Table S1 Studies included in this systematic review.pdf]

Supplementary Table S1. Studies included in this systematic review

Ref.	Type of study	Cell line	Animal model	miRNA	Intervention	Gene(s)/pathway regulated	Pharmacological effects
miRNA-inhibition therapy							
Alshehri 2021[26]	In vivo		CdCl2-induced NAFLD	miR-34a	inhibitor	SIRT1	Reduced hepatic fat accumulations in the livers
Bala 2021[79]	In vivo		HF-HC-HS induced NASH	miR-155	KO	NLRP3	Decreased steatosis, and attenuation in fibrosis
Chen 2020[76]	In vitro	UA treated AML-12 and HepG2 cells		miR-149-5p	inhibitor	FGF21	Ameliorated the triglyceride accumulation
Chen 2018[82]	In vivo and in vitro	FFA treated HepG2 cells and PH	HFD induced NAFLD	miR-1224-5p	inhibitor	AMPKα	Attenuated hepatic lipogenesis and steatosis
Cheng 2016[84]	In vitro	FFA treated treated Hepa 1-6 cells		miR-421	inhibitor	SIRT3, FOXO3	Decreased cellular oxidative damage
Chu 2022[67]	In vivo		HFD induced NAFLD	miR-103a-3p	inhibitor	HBP1	Decreased lipid accumulation, suppressed inflammatory response, reduced liver fibrosis
Csak 2015[80]	In vivo		MCD induced NASH	miR-155	KO	SMAD3, C/EBPβ	Reduce steatosis and fibrosis
Dai 2019[43]	In vivo		HFD induced NAFLD	miR-30b	antimiR	SERCA2b	Suppressed ER stress and insulin resistance
Ding 2015[27]	In vivo and in vitro	FFA treated L-02 cells	HFD induced NAFLD	miR-34a	inhibitor	PPARα, SIRT1	suppressed lipid accumulation and improved the degree of steatosis
Ding 2022[68]	In vivo and in vitro	FFA treated L-02 cells	HFD induced NAFLD	miR-103-3p	inhibitor	ACOX1	alleviated the accumulation of lipid droplets
Hanin 2018[87]	In vivo		MCD, CDE, HFHS or CDHFD induced NASH	miR-132	ASO	AM132	reversed the hepatic miR-132 excess and hyperlipidemic phenotype

Hu 2022[71]	In vivo and in vitro	PA treated L02 cells	HFD induced NAFLD	miR-122-5p	ASO	FOXO3	Attenuated inflammatory response and oxidative stress damage in dietary-induced NAFLD
Lee A 2021[28]	In vitro	FFA treated HepG2 cells		miR-34a-5p	inhibitor	NFE2L2	mediate cellular lipid accumulation
Lee D 2021[90]	In vivo		HFD induced NAFLD	miR-214-3p	antimiR	Ulk1	Mitigated hepatic steatosis
Lee Y 2021[19]	In vivo and in vitro	PA treated HepG2 and hu-7 cells	HFD or MCD induced NAFLD	miR-20b	antimiR	PPARα	Improved insulin sensitivity and a decrease in NAFLD progression
Lei 2018[91]	In vitro	PA treated HepG2 cells		miR-375	inhibitor	AdipoR2，TNF-α, IL-6	Up-regulated the expression of Adiponectin and inhibited the lipid accumulation
Liu 2020[23]	In vivo		HFD induced NAFLD	miR-130-5p	KO	IGFBP2, AKT	Prevented hepatic lipid accumulation and insulin resistance
Liu 2020[50]	In vivo and in vitro	PO treated PH	HFD induced NAFLD	miR-188	antimiR	Atg12	Alleviated diet-induced hepatosteatosis and insulin resistance
Long 2019[72]	In vitro	FFA treated HepG2 and Huh-7 cells		miR-122	inhibitor	SIRT1, LKB1/AMPK	Protected hepatocytes from lipid metabolic disorders
Ng 2014[97]	In vivo and in vitro	OA treated HepG2 cells and PH	HFD induced NAFLD	miR-24	ASO	Insig1	Impaired hepatic lipid accumulation and reduced plasma triglycerides
Nie 2017[98]	In vivo and in vitro	PA treated HepG2 cells	HFD induced NAFLD	miR-194	ASO	FXR, Nr1	Attenuated structural disorders, lipid deposits and inflammatory response
Riaz 2021[51]	In vivo and in vitro	LX-2 cells	HFD induced NAFLD	miR-188-5p	inhibitor	PTEN/AKT	Suppressed the HF parameters, pro-fibrotic and pro-inflammatory genes, and fibrosis
Rodigues 2017[64]	In vivo		MCD induced NASH	miR-21	KO	PPARα, FXR	Decreased steatosis, inflammation and lipoapoptosis, with impairment of fibrosis
Simino 2021[33]	In vitro	NEFA induced AML-12 cells		Let-7a	antimiR	AMPKα2	reduce fat accumulation

Sun 2022[99]	In vivo and in vitro	PA induced PH	AMLN induced NASH	miR-802	ASO	AMPK	Ameliorated steatosis, inflammation, apoptosis
Sun 2019[46]	In vitro	FFA induced HepG2 cells		miR-140	Knockdown	Neat1	Alleviated the severity of NAFLD
Wang G 2020[100]	In vitro	HFD mice isolated PH		miR-124-3p	inhibitor	Pref-1	Reduced triglyceride contents in hepatocytes and inflammatory factors
Wang L 2020[29]	In vivo and in vitro	OA and excess iron-treated HepG2 cells	Excess iron-induced NAFLD	miR-34a	antimiR	SIRT1	Alleviated triglyceride accumulation
Wang L 2016[81]	In vivo		HFD induced NAFLD	miR-155	inhibitor	LXRα	Decreased intracellular lipid content
Wang X 2019[65]	In vivo		MCD induced NAFLD	miR-21	antagomiR	LRP6, WNT/β-catenin	Alleviated lipid accumulation and inflammation
Wang Y 2017[105]	In vivo and in vitro	PA treated HepG2 cells	HFD induced NAFLD	miR-181b	inhibitor	SIRT1	Alleviated hepatic steatosis
Wu 2016[66]	In vivo and in vitro	OA treated HepG2 cells	HFD induced NAFLD	miR-21	ASO	HBP1-P53-Srebp1c	Reduced expression of genes controlling lipogenesis and cell cycle transition
Xu 2018[107]	In vivo and in vitro	FFA treated L-02 cells	HFD induced NAFLD	miR-190b	inhibitor	IGF-1, ADAMTS9	Suppressed lipid accumulation and improved insulin sensitivity
Xu 2021[31]	In vivo and in vitro	Kupffer cell	HFCF induced NASH	miR-34a	inhibitor	Cyp7a1, Cyp8b1, FAO	Reversed HFCF diet-induced steatohepatitis
Yu 2022[110]	In vivo and in vitro	FFA treated PH	HFD induced NAFLD	miR-665-3p	antagomiR	FNDC5/AMPKα	Prevented oxidative stress, inflammation and hepatic dysfunction
Zhang B 2019[32]	In vivo and in vitro	AML12	HFD induced NAFLD	miR-34a-5p	inhibitor	Mirt2, USP10	Attenuated hyperglycemia, insulin resistance and steatosis
Zhang Q 2021[45]	In vivo and in vitro	FFA treated HepG2 cells	HFD induced NAFLD	miR-125b	inhibitor	TNFAIP3, NF-κb	Ameliorated liver damage and inflammatory responses
Zhang T 2018[112]	In vivo and in vitro	OA treated HepG2 cells	HFD induced NAFLD	miR-378	ASO	Nrf1	Improved FAO and decreased hepatosteatosis

Zhuge 2017[49]	In vivo		HFD induced NAFLD	miR-150	KO	CFLAR	Regulated hepatic steatosis and insulin resistance
Zuo 2021[114]	In vivo and in vitro	PA treated HepG2 cells	MCD or HFD induced NAFLD	miR-7	inhibitor	LAMP1/CathepsinB/NLRP3	Improved endothelial barrier integrity
Ao 2016[35]	In vitro	OA treated L-02 cells		miR-9	inhibitor	Onecut2, SIRT1	Decreased intracellular lipid content
Chen X 2021[83]	In vitro	FFA treated L-02 cells		miR-380-5p	inhibitor	LRP2	Reduced lipid formation and accumulation
Li B 2014[55]	In vitro	FFA treated HepG2/AML12 cells		miR-199a-5p	antimiR	CAV1, PPARα	Alleviated deposition of FA and increased ATP levels and mtDNA contents
Wang J 2019[101]	In vitro	FFA treated L-02 cells		miR-222	antagomiR	ACOX1	Inhibited the accumulation of triglyceride
Xiao 2016[78]	In vitro	FFA treated HepG2 cells		miR-149	inhibitor	FGF21	Decreased lipogenesis
Yang 2021[108]	In vitro	FFA treated L-02 cells		miR-22	inhibitor	Sirt1	Reduced triglyceride levels without affecting cell activity
Zhang Q 2020[111]	In vitro	SA or PA treated AML12 cells		miR-295-5p	inhibitor	CAV1	Decreased hepatic lipid accumulation
Li Y 2022[56]	In vitro	FFA treated HepG2 cells		miR-200c	inhibitor	SIK2/PI3K/Akt	Decreased lipid accumulation
Liu H 2021[36]	In vivo and in vitro	PA treated L-02 cells	HFD induced NAFLD	miR-9-5p	antimiR	TGM2	Alleviated macrophage inflammation and M1 polarization and attenuated pathological change
Wang Y 2020[57]	In vivo		HFD induced NAFLD	miR-200	inhibitor	GRHL2, SIRT1, MAPK	Inhibited liver fibrosis and intestinal mucosal barrier dysfunction
Xiao J 2016[59]	In vitro	FFA treated HepG2 cells		miR-212	inhibitor	FGF21	Inhibited lipogenesis
Kong 2022[89]	In vitro	PA treated Hepa1-6 cells		mmu-miR-599	inhibitor	PXR	Inhibited steatosis, focal necrosis and inflammatory infiltration
Wang L 2022[102]	In vitro	PA treated L-02 cells		miR-138-5p	antimiR	PGC-1α	promoted lipid metabolism and inhibited inflammatory response

miRNA-replacement therapy							
Chai 2020[70]	In vivo		HFD induced NAFLD	miR-122	agomiR	RORA	Decreased fat droplets and total triglyceride content
Chen 2019[73]	In vitro	FFA treated HepG2 cells		miR-146a-5p	mimic	ROCK1	Inhibited lipid accumulation
Chen Z 2020[77]	In vivo and in vitro	HFD mice isolated liver cells	HFD induced NAFLD	miR-149	agomiR	ATF6	Alleviated ERS-induced inflammation and apoptosis
Cheng 2017[54]	In vivo and in vitro	FFA treated Hepa 1-6 cells	HFD induced NAFLD	miR-199a-3p	mimic	Sp1	Suppressed hepatic lipogenesis
Dong 2022[85]	In vivo and in vitro	PA treated PH and Huh7 cells	HFHC induced NAFLD	miR-379-5p	mimic	STAT1, HMGCS1	Reduced total cholesterol and free cholesterol and ameliorated hepatic injury reflected
Fan 2017[44]	In vivo and in vitro	PA treated HepG2 cells	db/db mice induced NAFLD	miR-30c-5p	mimic	FASN	Attenuated triglyceride accumulation and hepatic steatosis
Fan 2021[86]	In vivo and in vitro	FFA treated HepG2 cells	HFHFr induced NAFLD	miR-552-3p	mimic	LXRα, FXR	Inhibited metabolic gene expression in vitro and improved glycolipid metabolism
He 2017[38]	In vivo and in vitro	CD4 T cells	HFD induced NAFLD	miR-26a	pre-miR-26a	IL-6, IL-17	Decreased total liver weight, hepatic triglyceride deposition and serum ALT concentration
He 2018[74]	In vivo and in vitro	PA treated Hep G2 and AML-12 cells	MCD induced NASH	miR-146b	mimic	MyD88, PPARγ	alleviate the hepatic steatosis
He 2019[60]	In vitro	PA treated AML-12 cells		miR-223	mimic	Cxcl10, Tar	Controlled steatosis-to-NASH progression
Hu 2019[88]	In vivo and in vitro	OA treated HepG2 and PH cells	HFD induced NAFLD	miR-205	mimic	NEU1	Suppressed lipid accumulation
Hur 2015[62]	In vitro	PA treated HepG2 cells		miR-451	mimic	Cab39,AMPK/AKT	Prevented the progression from simple steatosis to severely advanced liver disease

Guo 2017[58]	In vivo and in vitro	Oleatic sodium treated PH	HFD induced NAFLD	miR-212-5p	mimic	FAS, SCD1	Decreased triglyceride accumulation
Li D 2015[92]	In vitro	Kupffer cells and BMM		miR-144	mimic	TLR2	Decreased proinflammatory cytokine production
Li H 2021[93]	In vivo		HFD induced NAFLD	miR-23b	agomiR	Acot4, ACC	Reduced liver weight and alleviated liver inflammation, apoptosis, and fibrosis
Li K 2020[75]	In vivo and in vitro	FFA treated AML-12 cells	HFD induced NAFLD	miR-146a	mimic	MED1	Improved lipid metabolism and mitochondrial function
Li P 2020[94]	In vitro	PA treated L-02 cells		miR-99a	mimic	NOX4	Reversed the pathological changes of liver
Lin 2019[42]	In vivo and in vitro	HepG2 cells	HFD induced NAFLD	miR-29a	transgene mice and mimic	PPARγ, CD36	Improved HDF-induced obesity, hepatocellular steatosis, and fibrosis
Lin 2017[52]	In vitro	Bisphenol A treated HepG2 cells		miR-192	mimic	SREBF1	Improved BPA-induced hepatic steatosis
Liu 2019[23]	In vitro	FFA treated HepG2 and Huh-7 cells		miR-130	mimic	PPARγ, CD36	Inhibited lipogenesis and TG secretion
Mittal 2020[96]	In vitro	PA treated HepG2 cells		miR-3666	overexpression	PPARγ	Decreased hepatic steatosis
Murata 2019[40]	In vitro	3T3-L1 cells		miR-27b	mimic	ACT2	Suppressed lipid accumulation and adipocyte differentiation
Niu 2022[61]	In vivo and in vitro	PA treated NCTC1469 cells	HFD induced NAFLD	miR-223-3p	mimic	E2F1	Suppressed lipid accumulation and liver fibrosis
Tang 2019[63]	In vitro	HFD mice isolated PH		miR-451	mimic	MIF, AKT	Suppressed Akt and promoted autophagy
Wang X 2020[37]	In vitro	FFA treated HepG2 cells and PH		miR-20a-5p	mimic	CD36	Reduced lipid accumulation
Wang X 2022[104]	In vivo and in vitro	FFA treated HepG2 and AML-12 cells	HFD induced NAFLD	miR-376b-3p	mimic	FGFR1	Ameliorated hepatic lipid accumulation
Wang Z 2020[53]	In vivo		HFD or HFrD induced NAFLD	miR-192-3p	overexpression	NR3C1	Controlled fat accumulation and insulin sensitivity in liver

Wolfson 2018[47]	In vitro	PA treated HepG2cells		miR-140	overexpression	TLR4/NFκB	Decreased inflammatory activity
Wu 2017[106]	In vivo and in vitro	FFA treated HepG2 cells and PH	HFD induced NAFLD	miR-206	mimic	Ptpn1	Facilitated insulin signaling and impairing hepatic lipogenesis
Xu 2021[39]	In vivo and in vitro	FFA treated HepG2 cells and PH	HFD induced NAFLD	miR-26a	overexpression	ER, EIF2α	Mitigated high-fat diet-induced ER stress and hepatic steatosis
Xu 2015[31]	In vivo and in vitro	FFA treated HepG2 cells and PH	HFD induced NAFLD	miR-34a	mimic	HNF-4α	Inhibited very low-density lipoprotein secretion and promotes liver steatosis and hypolipidemia
Yang 2020[119]	In vivo and in vitro	HepG2 cells	MCD induced NASH	miR-29a	mimic	GSK3β, SIRT1	Inhibited mitochondrial biogenesis and alleviated proteostatic stress and UPRmt
Yu 2021[109]	In vivo and in vitro	FFA treated PH	HFD induced NAFLD	miR-137-3p	agomiR	AMPKα	Alleviated oxidative stress, inflammation, and hepatic dysfunction
Zhang M 2017[120]	In vivo and in vitro	OA treated PH	HFD induced NAFLD	miR-27a	mimic	Fasn, Scd1	Reduced liver TG contents
Zhang M 2020[69]	In vivo and in vitro	OA treated PH	HCD induced NAFLD	miR-103	overexpression	Fasn, Scd1	Inhibited hepatic DNL to repress HCD-promoted hepatic lipid contents
Zhang Z 2015[121]	In vivo and in vitro	FFA treated HepG2 cells and PH	HFD induced NAFLD	miR-125b	mimic	FAS, Estrogen	Inhibited liver steatosis
Wang Y 2017[22]	In vitro	HSC-T6		miR-130a-3p	mimic	TGF-β/SMAD, TGFBR1, TGFBR2	Attenuated activation and induced apoptosis of hepatic stellate cells
Zhao 2022[34]	In vitro	PA treated PH		Let-7b-5p	mimic	TGF-β	Reduced mitochondrial oxidative phosphorylation and suppressed white-to-beige fat conversion
Guo 2022[25]	In vitro	FFA treated HepG2 cells		miR-130b-3p	mimic	RCOK1, HOTAIR	Decreased lipid accumulation
Li R 2022[95]	In vitro	HG and FFA treated Hepa1-6 cells		miR-5120	mimic	ABHD6	Improved liver function and hepatic steatosis

Zhang N 2021[48]	In vitro	FFA treated AML-12 cells	miR-150	mimic	CXCR4	Mitigated HFD-induced NAFLD
Zhang Y 2020[113]	In vitro	PA treated HepG2 cells	miR-96-5p	agomiR	P66shc	Alleviated ROS, apoptosis and hepatic steatosis
Wang X 2018[103]	In vitro	FFA treated AML-12 cells	miR-136	mimic	MEG3, Nrf1	Decreased serum lipid level and alleviated NAFLD

OA: Oleic acid; UA: Urine acid; SA: Stearic acid; PA: Palmitic acid; FFA: Free fat acid; PH: Primary hepatocytes; NAFLD: Non-Alcoholic Fatty Liver Disease; NASH: Non-alcoholic steatohepatitis; HFD: High-fat diet; MCD: Methionine-choline deficient; HF-HC-HS: High fat-high cholesterol-high sugar-diet; HFHS: High-fat-high-sucrose; CDHFD: Choline-deficient high fat diet; HFHC: High-fat and high-cholesterol; HFHFr: High-fat and high-fructose; HFCF: high fat, cholesterol, and fructose; CDE: Choline-deficient, ethionine-supplemented; AMLN: Amylin liver NASH;

KO: Knockout; ASO: Antisense oligonucleotides; NEFA: Non-esterified fatty-acids
