# Supplementary material for: Therapeutic Effects of microRNAs on Nonalcoholic Fatty Liver Disease (NAFLD) and Nonalcoholic Steatohepatitis (NASH): A Systematic Review and Meta-Analysis
Source: Int J Mol Sci. 2023 May 23;24(11):9168. doi: 10.3390/ijms24119168 (PMC10252239; doi:10.3390/ijms24119168)
Supplement: Supplementary file 1 [file ijms-24-09168-s001.zip › Supplementary Table S2 Models and treatment results are included in this systematic review.pdf]

Table S2.Models and treatment results are included in this systematic review

Ref.	Country/Region	Animal/Gender	Intervention	Biological processes
miRNA-inhibition therapy				
Alshehri 2021[26]	Saudi Arabia/Asia	rats/male	NAFLD induction	Fat accumulation
Bala 2021[79]	America/North America	C57BL/6、miR-155 KO mice/male	NAFLD induction	Fat accumulation、Fibrosis
Chen 2020[76]	China/Asia			Fat accumulation
Chen 2018[82]	China/Asia	C57BL/6 mice/male	Post NAFLD	Fat accumulation
Cheng 2016[84]	China/Asia			Oxidative stress
Chu 2022[67]	China/Asia	C57BL/6J mice/male	Post NAFLD	Fat accumulation、Inflammation、Fibrosis
Csak 2015[80]	America/North America	C57BL/6、miR-155 KO mice/female	NASH induction	Fat accumulation、Fibrosis
Dai 2019[43]	China/Asia	rats/male	Post NAFLD	Oxidative stress、Fibrosis、Insulin resistance
Ding 2015[27]	China/Asia	C57BL/6 mice/male	Post NAFLD	Fat accumulation
Ding 2022[68]	China/Asia	C57BL/6 mice/male	Post NAFLD	Fat accumulation
Hanin 2018[87]	Israel/Asia	C57BL/6J mice/male	Post NASH	Fat accumulation

Hu 2022[71]	China/Asia	C57BL/6J mice/male	Post NAFLD	Oxidative stress、Inflammation
Lee A 2021[28]	China/Asia			Fat accumulation
Lee D 2021[90]	South Korea/Asia	C57BL/6J mice/male	Post NAFLD	Fat accumulation
Lee Y 2021[19]	South Korea/Asia	C57BL/6J mice/male	NAFLD induction	Insulin resistance
Lei 2018[91]	China/Asia			Fat accumulation
Liu 2020[23]	China/Asia	C57BL/6 mice/male	Post NAFLD	Fat accumulation、Insulin resistance
Liu 2020[50]	China/Asia	C57BL/6J、db/db mice/male	NAFLD induction	Fat accumulation、Insulin resistance
Long 2019[72]	China/Asia			Fat accumulation
Ng 2014[97]	Singapore/Asia	C57BL/6mice/male	Post NAFLD	Fat accumulation
Nie 2017[98]	China/Asia	C57BL/6J mice/male	Post NAFLD	Fat accumulation、Inflammation
Riaz 2021[51]	China/Asia	C57BL/6J、E3 rasts mice/male	NAFLD induction	Inflammation、Fibrosis
Rodigues 2017[64]	Portugal/Europe	C57BL/6、miR-21 KO mice/male、female	NASH induction	Fat accumulation 、 Inflammation 、 Fibrosis 、 Other （Apoptosis）
Simino 2021[33]	Brazil/South America			Fat accumulation

Sun 2022[99]	Israel/Asia	C57BL/6 mice/male	Post NASH	Fat accumulation、Inflammation、Other（Apoptosis）
Sun 2019[46]	China/Asia			Fat accumulation
Wang G 2020[100]	China/Asia			Fat accumulation、Inflammation
Wang L 2020[29]	China/Asia	C57BL/6 mice/male	NAFLD induction	Fat accumulation
Wang L 2016[81]	China/Asia	C57BL/6J mice/Unclear	Post NAFLD	Fat accumulation
Wang X 2019[65]	China/Asia	C57BL/6J mice/male	Post NAFLD	Fat accumulation、Inflammation
Wang Y 2017[105]	China/Asia	C57BL/6 mice/male	NAFLD induction	Fat accumulation
Wu 2016[66]	America/North America	C57BL/6 mice/male	Post NAFLD	Fat accumulation
Xu 2018[107]	China/Asia	C57BL/6 mice/male	Post NAFLD	Fat accumulation、Insulin resistance
Xu 2021[31]	America/North America	C57BL/6J mice/male	NAFLD induction	Fat accumulation、Inflammation、Fibrosis、Other（Apoptosis）
Yu 2022[110]	China/Asia	C57BL/6 mice/male	Post NAFLD	Inflammation、Oxidative stress
Zhang B 2019[32]	China/Asia	C57BL/6、ob/ob、db/db mice/male	Post NAFLD	Fat accumulation、Insulin resistance
Zhang Q 2021[45]	China/Asia	C57BL/6、miR-125b KO mice/male	Post NAFLD	Inflammation

Zhang T 2018[112]	America/North America	C57BL/6 mice/male	Post NAFLD	Fat accumulation
Zhuge 2017[49]	China/Asia	C57BL/6J、miR-150 KO mice/male	NAFLD induction	Fat accumulation、Insulin resistance
Zuo 2021[114]	China/Asia	C57BL/6N mice/male	Post NAFLD	Other（Endothelial Barrier Integrity）
Ao 2016[35]	China/Asia			Fat accumulation
Chen X 2021[83]	China/Asia			Fat accumulation
Li B 2014[55]	China/Asia			Fat accumulation、Oxidative stress
Wang J 2019[101]	China/Asia			Fat accumulation
Xiao 2016[78]	China/Asia			Other（Lipogenesis）
Yang 2021[108]	China/Asia			Fat accumulation、Inflammation
Zhang Q 2020[111]	China/Asia			Fat accumulation
Li Y 2022[56]	China/Asia			Fat accumulation
Liu H 2021[36]	China/Asia	C57BL/6J mice/male	Post NAFLD	Fat accumulation、Inflammation
Wang Y 2020[57]	China/Asia	C57BL/6 mice/Unclear	Post NAFLD	Fat accumulation、Fibrosis、Other（Intestinal mucosal barrier dysfunction）

Xiao J 2016[59]	China/Asia			Other（Lipogenesis）
Kong 2022[89]	China/Asia			Fat accumulation、Inflammation、Other（Necrosis）
Wang L 2022[102]	China/Asia			Fat accumulation、Inflammation
miRNA-replacement therapy				
Chai 2020[70]	Israel/Asia	C57BL/6 mice/male	Post NAFLD	Oxidative stress、Other（Lipogenesis）
Chen 2019[73]	China/Asia			Fat accumulation
Chen Z 2020[77]	China/Asia	C57BL/6 mice/male	Post NAFLD	Inflammation、Other（Apoptosis）
Cheng 2017[54]	China/Asia	C57BL/6J mice/male	Post NAFLD	Fat accumulation、Other（Lipogenesis）
Dong 2022[85]	China/Asia	C57BL/6J、db/db mice/male	Post NAFLD	Other（Lipotoxicity）
Fan 2017[44]	China/Asia	C57BL/6Ks、db/db mice/male	Post NAFLD	Fat accumulation
Fan 2021[86]	China/Asia	HFHFr、db/db mice/Unclear	Post NAFLD	Other（Glucolipid metabolism）
He 2017[38]	China/Asia	C57BL/6 mice/male	Post NAFLD	Fat accumulation、Inflammation
He 2018[74]	China/Asia	C57BL/6 mice/female	Post NASH	Fat accumulation、Inflammation
He 2019[60]	America/North America			Inflammation、Fibrosis、Oxidative stress

Hu 2019[88]	China/Asia	C57BL/6 mice/male		Fat accumulation
Hur 2015[62]	China/Asia			Inflammation
Guo 2017[58]	China/Asia	C57BL/6J mice/male	Post NAFLD	Fat accumulation
Li D 2015[92]	China/Asia			Inflammation
Li H 2021[93]	China/Asia	C57BL/6、miR-23b KO、db/db mice/male	Post NAFLD	Fat accumulation、Inflammation、Fibrosis、Other（Apoptosis）
Li K 2020[75]	China/Asia	C57BL/6 mice/male	Post NAFLD	Other（Glucolipid metabolism）
Li P 2020[94]	China/Asia			Oxidative stress、Fibrosis、Other（Glucolipid metabolism）
Lin 2019[42]	China/Asia	C57BL/6J、miR-29a TG mice/male	NAFLD induction	Fat accumulation、Fibrosis
Lin 2017[52]	China/Asia			Fat accumulation、Other（Glucolipid metabolism）
Liu 2019[23]	China/Asia			Fat accumulation、Other（Lipogenesis）
Mittal 2020[96]	India/Asia			Fat accumulation
Murata 2019[40]	Japan/Asia			Fat accumulation
Niu 2022[61]	China/Asia	C57BL/6J mice/female	NAFLD induction	Fat accumulation、Fibrosis

Tang 2019[63]	China/Asia			Other（Autophagy）
Wang X 2020[37]	China/Asia			Fat accumulation
Wang X 2022[104]	China/Asia	C57BL/6 mice/male	NAFLD induction	Fat accumulation
Wang Z 2020[53]	China/Asia	C57BL/6、ob/ob、db/db mice/male	NAFLD induction	Fat accumulation、Insulin resistance
Wolfson 2018[47]	America/North America			Inflammation
Wu 2017[106]	America/North America	C57BL/6 mice/male	Post NAFLD	Fat accumulation、Insulin resistance
Xu 2021[39]	China/Asia	C57BL/6、miR-26a TG mice/male	NAFLD induction	Fat accumulation、Oxidative stress
Xu 2015[31]	America/North America	C57BL/6、ob/ob、db/db mice/male	Post NAFLD	Fat accumulation
Yang 2020[119]	China/Asia	C57BL/6、miR-29a TG mice/male	NASH induction	Oxidative stress
Yu 2021[109]	China/Asia	C57BL/6 mice/male	Post NAFLD	Inflammation、Oxidative stress
Zhang M 2017[120]	China/Asia	C57BL/6J mice/male、 ob/ob mice/male	Post NAFLD	Fat accumulation、Other（Lipogenesis）
Zhang M 2020[69]	China/Asia	C57BL/6J mice/male、db/db mice	Post NAFLD	Fat accumulation
Zhang Z 2015[121]	China/Asia	C57BL/6J mice/female	NAFLD induction	Fat accumulation、Other（Lipogenesis）

Wang Y 2017[22]	China/Asia	Fibrosis、Other（Apoptosis）
Zhao 2022[34]	China/Asia	Other（Oxidative phosphorylation）
Guo 2022[25]	China/Asia	Fat accumulation
Li R 2022[95]	China/Asia	Fat accumulation
Zhang N 2021[48]	China/Asia	Fat accumulation、Inflammation、Insulin resistance
Zhang Y 2020[113]	China/Asia	Fat accumulation、Oxidative stress、Other（Apoptosis）
Wang X 2018[103]	China/Asia	Fat accumulation、Other（Lipogenesis）

KO: Knockout; TG:Transgenic

NAFLD: Non-Alcoholic Fatty Liver Disease; NASH: Non-alcoholic steatohepatitis; HFD: High-fat diet; MCD: Methionine-choline deficient; HF-HC-HS: High fat-high cholesterol-high sugar-diet;
